# An overview on ethical considerations in stem cell research in Iran and ethical recommendations: A review

**Published:** 2017-02

**Authors:** Tahmineh Farajkhoda

**Affiliations:** *Research Center for Nursing and Midwifery Care, Shahid Sadoughi University of Medical Sciences, Yazd, Iran.*

**Keywords:** Cell therapy, Embryo, Ethics, Stem cells, Recommendation, Iran

## Abstract

Conducting research on the stem cell lines might bring some worthy good to public. Human Stem Cells (hSCs) research has provided opportunities for scientific progresses and new therapies, but some complex ethical matters should be noticed to ensure that stem cell research is carried out in an ethically appropriate manner. The aim of this review article is to discuss the importance of stem cell research, code of ethics for stem cell research in Iran and ethical recommendation. Generation of stem cells for research from human embryo or adult stem cells, saving, maintenance and using of them are the main ethical, legal and jurisprudence concerns in Iran. Concerns regarding human reproduction or human cloning, breach of human dignity, genetic manipulation and probability of tumorogenisity are observed in adult/somatic stem cells. Destruction of embryo to generate stem cell is an important matter in Iran. In this regards, obtaining stem cell from donated frozen embryos through infertility treatment that would be discarded is an acceptable solution in Iran for generation of embryo for research. Ethical, legal, and jurisprudence strategies for using adult/somatic stem cells are determination of ownership of stem cells, trade prohibition of human body, supervision on bio banks and information of Oversight Committee on Stem Cell Research. Recommendations to handle ethical issues for conducting stem cell research are well-designed studies, compliance codes of ethics in biomedical research (specifically codes of ethics on stem cell research, codes of ethics on clinical trials studies and codes of ethics on animals studies), appropriate collaboration with ethics committees and respecting of rights of participants (including both of human and animal rights) in research. In addition, there is a necessity for extending global networks of bioethics for strengthening communications within organizations at both the regional and international level, strengthening legislation systems, designing and establishing convenient collaborative educational courses at different levels.

## What is stem cell?

The word ‘stem cell’ by itself has been association with complexity (1). Patients, scientists, and the public, on hearing the term ‘stem cell’, may be confused for reason that they cannot separate experimental stem cell interventions from proven stem cell therapies (2-8). Stem cells are considered as unspecialized cells that they have ability to divide and create copies of themselves and having the potential to differentiate, for instance to produce other human cell types in the body (9). These cells could be obtained from the embryo, cord blood, the fetus or the adult cells. Using of surplus embryos (at the blastocyst stage) in in vitro fertilization (IVF) treatment programs, is another way for generation stem cells in the human. In addition stem cells may be produced from donated gamets, frozen embryos or cloned embryos in pathway of generation somatic cell nuclear transfer (SCNT) (10-13). Ovarian tissue of fetuses, children and pre- and post-menarchal women are considered as other ways for obtaining immature oocytes (10).

Several highly multipotent stem cells for example mesenchymal stem/stromal cells and stem cells obtained from amniotic fluid, umbilical cord blood, adipose tissue, or urine providing the occasion for widespread bio banking and increasing availability (1). Pluripotent stem cell lines can be obtained from the inner cell mass of the 5-7 days old human blastocyst (12, 14). It must be taken into account that, whereas human emberyonic stem cells (hESCs) and human amniotic fluid stem cells (hAFSCs) are naturally existing stem cell, human induced pluripotent stem cells (hiPSCs) (and also SCNT-hESCs) are artificially generated (14, 15).

## History of stem cell research

Reports concerning carry out of studies on the use of animal embryonic stem cells have been published since the early 1980s. Cell differentiation research and therapeutic use of embryonic stem cells in order to renew tissues in a wide range of serious, but common, diseases have been reported (10). In 1998, scientists discovered that embryonic stem cells could be obtained from early human embryos (1, 16). In February 2004, South Korean researchers announced that they are the first investigators in the world to successfully produce stem cells and create a stem cell line from a cloned human embryo (17). 

Over the last 20 years a revolution has been happened in researchers’ ability to produce stem cells from several sources (18). A committee to develop systematic guidelines for research centers and investigators who interested in study in the field of hESC research was formed by the National Academy of Sciences in 2003 (19). According to aforementioned guidelines that were revised in 2010, permitted and unpermitted categories of hESC research was determined carefully and additionally formation of embryonic stem cell research oversight committees (ESCROs) in order to supervise of research review process was recommended (1, 20). 

## The importance of stem cell research

Stem cell research is considered as a surprising but complex and controversial science. Stem cell-based researchers have considerable attention in this filed, since stem cell publications overall are cited 50% more than all other publications in related fields (21). This research has potential to change the treatment of human diseases, and provide an applicable approach to invest greatly in stem cell research (21, 22). Still, human stem cells (hSCs) research has been associated with various ethical and regulatory issues that influence on a nation’s policies (21-24). 

The United States is considered as leader in stem cell research since that they publish the highest articles focus on stem cell research with high field-weighted citation impact. Recently, stem cell research has risen extraordinarily, showing a growing rate more than double the rate of world research publications from 2008-2012. Nevertheless, this growth is not equal across all stem cell research fields, for instance citation of induced pluripotent stem (iPS) cell publications is approximately three times the global rate in compare with ES cell publications. Nearly half of all stem cell publications are related to regenerative medicine or drug development using iPS cell research (21). 

The main purposes of hSCs research are genetic disorders modeling and stem cell-based human therapies and tissue or organ transplantation (12, 14). Human stem cells provide very crucial models for practical studies of genes involved in the molecular development of human genetic disorders (14). Because of having the ability of differentiation to produce other cell types, there is possibility to use them in regenerative medicine. Embryonic stem cells in compare with stem cells obtained from certain adult cells have enormous therapeutic potential because they can transform to every cell type in the human body. For this reason researchers have been interested to study embryonic stem cell (9, 25).

Exploration pharmacogenomics and development of organoids and other products for use in regenerative medicine are other usages of stem cells research (1, 12, 21-30). Stem cells can be used as sources for modeling different diseases, screening and drugs selection, regenerative medicine, and also introduce treatment via grafting the cells. This technology has been nearly successful in treatment of different medical fields. A hopefully progress has been reported for new treatments for diseases such as diabetes, spinal cord injury, Parkinson’s disease, and myocardial infarction by stem cells in USA (12). In addition, tumor therapy has been introduced in China and also in USA (31, 32). In Iran great advances in tissue engineering for vascular grafts, prognosis and treatment of breast cancer and acute kidney injury has been observed (33-35).

## Ethical concerns regarding sources of stem cells

The ethical implications of sources of stem cells are complex and controversial (10). Discovery of multipotent stem cells like human embryonic stem cells has emerged new ethical and political challenges in stem cell research (1, 36-38). The ethical concerns have been raised from destruction of embryo for obtaining multipotent stem cells. IPS cells are functionally similar to ESCs (29). Although it seems that iPS cells do not have the ethical problems related to the use of embryonic stem cells, but there are several ethical dilemma (12). The genetic manipulations for derivation iPS cells may increase the risks of toxicity, tumorigenicity and immunological reactions (1, 29).

## Stem cell research in Iran

All the contemporary ethical principles and SRH rights are highlighted in Islamic Shariaa. SRH care services should be provided for Muslim families or communities. Islam always encouraged scientific research, especially research on finding cures for human disease. Therefore human embryonic stem cell research is supported and permitted for the reason of potential benefits (24). In Iran embryo research has been influenced by the religious, cultural and social opinions concerning when human life is begun (22). 

It should be noticed that bioethics researches in Islamic countries have been joined to religious jurisprudence. Ethical issues concerning stem cell research are debated among medical physicians and religious scholars (39). In the Holley Qur`an embryological development of fetuses has been carefully and uniquely mentioned (40, 41). The main question is raised from whether we allow destroying one life (destruction of the embryo) to save another one or we should close our eyes to the potential of life-saving therapies out of respect for the potential life of the embryo as a person? Producing human embryos only for research purposes is illegal. Additionally, only the use of human embryos below 14 days that were created through IVF techniques, but which are not inserted into the uterus are considered ethically because the differentiation of the blastocyst is initiated on the day 14 (42, 43). The majority of Muslim scholars also agree with therapeutic cloning by the means of use supernumerary early pre-embryos which are produced through IVF treatment. The early pre-embryos are regarded in Islamic jurisprudence as worthy of respect but do not have the full sanctity offered to the embryo after implantation in the uterus and especially after ensoulment (24). 

**Figure 1:**
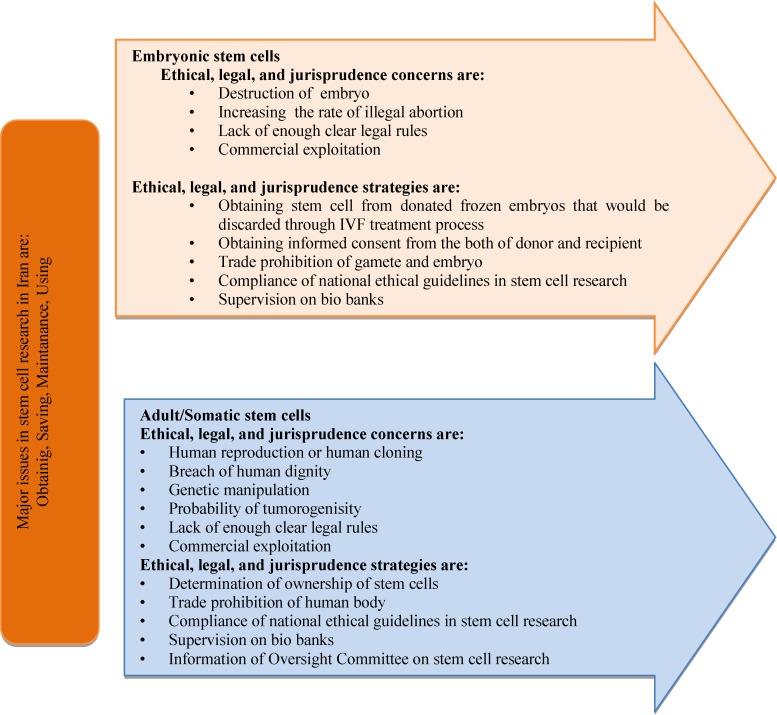
Ethical, legal and jurisprudence concerns based on the origin of stem cell in Iran

According to Islamic view ensoulment is happened at 120 days after fertilization (whereas a minority of Muslims believe that it is occurred 40 days after conception) (44). Based on another Islamic opinion, the embryo in the pre-ensoulment phases is alive and its eradication is considered as a sin and criminal action. Totally according to Shi`ite opinion there is little problem concerning research on stem cells based on national ethical guidelines that improves potential therapeutic value (45, 46). The Shiaa sect permits egg donation (40). In Islamic perspective there is difference between reproductive and therapeutic cloning and stem cell research (22). In 2002, human embryonic research was led to produce the stem cells in Iran (16). The major purpose of this investigation was to realize human cell differentiation and developmental biology and to generate specialized cells to treat many of diseases and conditions (47). In Figure 1, ethical, legal, and jurisprudence concerns have been illustrated based on the origin of stem cell in Iran (43, 48-52). 

In the recent years, considerable advances have been happened in biomedical ethics, particularly in the field of education, research and legislation in Iran. The establishment of the National and Regional Committees for Medical Research Ethics, the development of national codes of ethics in biomedical research in the 1990s, then the first, the second and the third revision of them in 2002, 2005 and 2014 respectively and also the introduction of a comprehensive strategic plan for medical ethics at the national level in 2002 were valuable activities by the Ministry of Health and Medical Education (MOHME) (44, 53-56).

An over view on the responsibility of ethics committees in research shows research ethics committees in general play an important role in evaluating the ethical aspects of medical research. A ‘Research Ethics Committee' (REC) is defined as a multidisciplinary, independent, body charged with reviewing research involving human participants to guarantee that their dignity, rights and welfare are protected, through reviewing research proposals, monitoring the conduct of research and dealing with problematic issues that may arise from research. They should be familiar with biomedical research principles including status and duties of research ethics committees and consequently strengthen the RECs in Iran (57). 

RECs activate in universities of medical sciences at the national, regional and local level. Although in Iran, Council of Policy Making in Medical Ethics and National Committee of Medical Ethics work on biomedical ethics issues but stem cell research centers and institutes have selected and formed their REC members and work under supervision of research deputy by selves. Lack of specific REC for stem cell research and oversight committee on stem cell research in Iran are obviously challenge that need to be resolved.

## National codes of ethics for stem cell research in Iran

Human dignity and health related issues, moral status of embryo and participants safety have especial value in Iranian culture (53-58, 59). Every researcher interested on stem cells research must compliance national codes of ethics in biomedical research, specific codes of ethics for research on stem cells, specific codes of ethics for research on animals and other related rules and regulations and existing related laws. In Iran, the final version of national codes of ethics in biomedical research (2014) including 19 articles regarding stem cell research is available in MOHME web site (53). 

Article 1: Authorized sources for producing pluripotent stem cells are 1) supernumerary embryos from IVF treatment programs (only with the purpose of infertility treatment), 2) aborted embryos, 3) induced embryos from research-therapeutic simulation, 4) IPs, 5) cord blood or placenta of delivered newborn.

Article 2: Researchers should use only the surplus embryos that they produce in order to treat infertility at the beginning. 

Article 3: Donated embryos for research must not be implanted into uterus of another women or another species of animals.

Article 4: Oocyte, sperm, embryo, fetus or any human biological material should not be obtained from commercial means.

Article 5: Freely informed consent should be gotten from both parents of embryo or fetus and third party who have donated the gamete in the time of donation for use of final embryo in research. 

Article 6: Infertility therapists should not be as a member of researchers group who investigate on stem cells. 

Article 7: Use of fetus for obtaining of stem cells should not have any impact on decision concerning abortion. In the case, physicians who decide to terminate the pregnancy should not be as one of research team members.

Article 8: Producing of oocyte is forbidden in order to generate stem cells, but use of supernumerary oocytes after infertility treatments are acceptable. 

Article 9: IPs should not be donated through research process: 1) Even though in autologous donation, 2) They should not be combined with human or non-human embryo and 3) They should not be added to human or non-human embryo. 

Article 10: Freely informed consent should be obtained from all participants in research. 

Article 11: Specific freely informed consent is mandatory from all participants in research based on type of research. 

Article 12: All needed information should be given for decision making to participate in the research in order to get freely informed consent, including: 1) explanations regarding type of using from donated human biological materials, 2) destruction probability of donated human biological materials across producing stem cells, 3) possibility of long-term saving of donated human biological materials in order to use of them in the future, 4) probability of economic interests resulted from research in present or in the future, 6) medical and therapeutic benefit resulted from research are not limited to donor and the public may use these benefits, 7) rejection of donors to participate in the research has not any impact on their treatment process, 8) donors can exclude from the research any time they would like to withdraw from participation in the research without any influence on their treatment, 9) donated embryo is not used in order to treat another couples with infertility, 10) necessity of conducting genetic and infection diseases screening tests by donor, 11) possibility of genetic changes in donated biological materials, 12) commercialization probability of donated biological materials without any right of donor for use it.

Article 13: In order to respect participants’ right of confidentiality all individual information should be kept in confidence.

Article 14: Research centers and treatments should provide necessary considerations to respect participants’ right of confidentiality (coding their records), even if/ though if they cannot provide them they should trash these information.

Article 15: Avoiding obtaining and saving of unnecessary information of participants.

Article 16: Research institutes should conduct enough pre-clinical research on animal models. 

Article 17: Small animal models should be used in order to 1) examination of wild type stem cells bond or ill or genetic modification, 2) assessment of healing after cell therapy, 3) investigation of tissue recovery mechanism, 4) assessment of diseases mechanism for effectiveness, age and degree, 5) assessment of rate and pathway of cell therapy. 

Article 18: Big animals models should be used in research that small animal models are not enough and research on structural tissues such as bone, tendon or cartilage.

Article 19: Research on primates should be conducted in the condition of necessity for providing the information that is not available from other ways. These researches should be directly supervised by an expert zoologist (53).

## Strengthnesses and weaknesses of national codes of ethics for stem cell research

**Strengthnesses**

The main principles of global bioethics (autonomy, beneficence, non-maleficence and justice) are acceptable in Islamic opinion (39, 58-60), but interpretation and application of them are different in Islamic jurisprudence. The main principles of Islamic ethics are the respect for human dignity, eternity of life, altruism and relation between human being with God and the universe (39, 61). Several Islamic principles for judgment on bioethical issues have been introduced by Islamic experts in related disciplines, including the principle of “the Public Interest” (Maslaha), the principle of “Do no Harm” (La Dararwa la Derar), the principle of “Necessity” (Darura), and the principle of “No Hardship” (La Haradj). Establishment of the aforementioned Islamic principles helps to present laws due to newly emerged ethical problems and they can solve clinical bioethics matters and research (62). 

In addition, Iranian investigators can use the holy Quran recommendations, principles of Islamic ethics; the religious perspectives (Fatwa) of Islamic experts on special problems; the national laws or ethics codes; international guidelines; and the norms of the society. Dynamic nature of Islamic bioethics helps investigators to conduct their research based on the modern innovations in scientific fields, such as the principles of “the public interest” (Maslaha) and “do no harm” (La Dararwa la Derar) (63, 64). 

In Iran, several laws related to bioethics were approved by the parliament legislation system in order to be used and compliance in the clinical practice by bioethics researchers (39). 

In Iran, developing codes of bioethics in research including stem cell research have been provided by specialists’ expert in different related fields of philosophy, law, jurisprudence, medicine and other related sciences. Therefore Islamic values and cultural norms including goods of public and individuals have been anticipated in development of the aforementioned codes (62). It seems that in design of national codes of bioethics in stem cell research both of global principles of bioethics research and Islamic viewpoints have been well considered and respected by Iranian developer team. 

For instance articles 1, 2, 3, 4, 8, 9, 15, 16 aforementioned codes have been written based on “Do no Harm” or “La Darar” principle. In articles 5, 7, 10, 11, 12, 13, 14 principle of autonomy has been considered. Principle of justice has been taken into account in article 6 and principle of beneficence has been noticed in articles 17, 18, 19. In addition, in developing of the codes major issues on participants, rights in research such as informed consent, confidentiality, decreasing the risks and harms to the possible minimal level during research process, reducing of conflict of interest and patenting issue and even animals’ rights in research have been anticipated and respected.

## Weaknesses

Although the establishment of the national codes of ethics in biomedical research including stem cell research in Iran has been an appreciable effort but it seems that these codes have not enough clarity, comprehensiveness and precise (58, 59, 65, 66). In addition some of these codes are behind of ethics domain and they are related to technical or law field (66). Although within the specific section on clinical trials, some topics have been mentioned including participants’ rights and informed consent, controlled trials, ethical review committee’s responsibilities and compensation issue, but it seems that informed consent should be more paid attention and highlighted. Need to develop a unique specific informed consent regarding stem cell research and establishment of it in MOHME website is a necessity. 

In addition, protection of confidentiality right of participant’s information has been mentioned generally and necessary details has not been considered such as duration, type of protecting system, level of confidentiality protection and accessibility, education of research team members and staffs. Risks management should be discussed more. Although compensation payments has been considered in the clinical trials codes of ethics but the role of health insurance system, length of compensation payments, approval process regarding types of risks and harms that induced by research itself should be mentioned with more clarity. Increasing interrelation of medicine, religion, ethics and law requires greater understanding and analysis of medical ethics issues and the provision of culturally-adapted solutions (39).

## Ethical recommendations on stem cell research in Iran

Ethical recommendations for stem cell researchers are well-designed studies, appropriate collaboration with ethics committees and respecting of human rights in research (1, 10, 12, 58, 59, 65, 67). Recommendations for bioethics empowerment and legislation are formulated for revision and updating of the national ethical guidelines for biomedical research based on new needs of related societies and public, better collaboration in national level as a teamwork cooperation including religious scholars, physicians, philosophers, legal experts, sociologists and other interested academics. It should be noticed that the religious opinions (Fatwa) of Islamic scholars on special issues provides appropriate Islamic culturally-adapted solutions for arguable matters based on Islamic views and public norms and values. Also strengthens of regional and international level collaboration on stem cell research such as sharing the findings of research, education and scholarly activities are recommended (39, 54, 55).

## Conclusion

Advancement in medical knowledge and practice is linked to stem cell research especially in reproductive medicine. Stem cells research is an inevitable necessity and the usefulness of stem cell technology for combating many diseases as well as to improve fertility treatment has been proved. In Iran, Islamic perspective supports scientists to conduct well-designed and ethically accepted research on finding cures for human diseases. Though human embryonic stem cell research has been permitted for the reason of potential benefits but human cloning has been forbidden. 

Recommendations to handle ethical issues for conducting stem cell research are well-designed studies, appropriate collaboration with ethics committees and respecting of rights of participants (including both of human and animal rights) in research. In addition, appropriate collaboration is needed at national and international level to extend global networks of bioethics for strengthening communications within related organizations. This article has focused on main ethical, legal and jurisprudence concerns regarding stem cell research in Iran and has provided applicable suggestions based on Iranian Islamic norms and values. In addition the strengthnesses and weaknesses analysis of national codes of ethics for stem cell research may encourage bioethics codes developers attempt for better modification, adjusting and revision of national valuable codes of ethics for stem cell research in Iran. Resolving of ethical, legal and jurisprudence concerns and challenges help Iranian stem cell researcher and scientists to produce and publish valuable and credible findings and introducing new products and drugs for marketing and treatment of serious diseases.
